# Correction: Comparative analysis of the complete chloroplast genome of Papaveraceae to identify rearrangements within the *Corydalis* chloroplast genome

**DOI:** 10.1371/journal.pone.0320969

**Published:** 2025-03-21

**Authors:** Sang-Chul Kim, Young-Ho Ha, Beom Kyun Park, Ju Eun Jang, Eun Su Kang, Young-Soo Kim, Tae-Hee Kim, Hyuk-Jin Kim

The seventh author’s name is spelled incorrectly. The correct name is: Tae-Hee Kim.
