# Supplementary figures and images for: Comparative analysis of the complete chloroplast genome of Papaveraceae to identify rearrangements within the Corydalis chloroplast genome
Source: PLoS One. 2023 Sep 21;18(9):e0289625. doi: 10.1371/journal.pone.0289625 (PMC10513226; doi:10.1371/journal.pone.0289625)

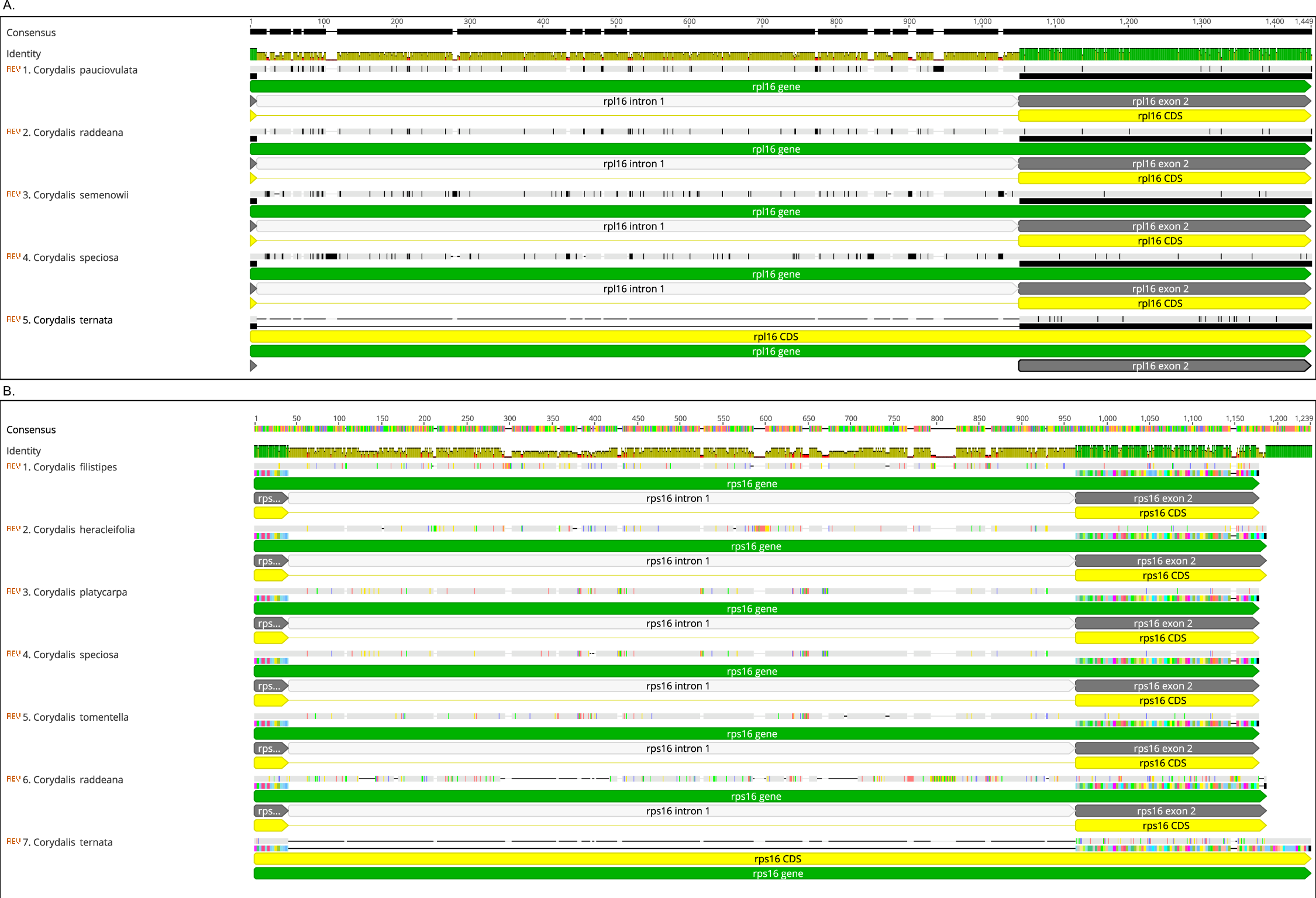

Supplement: S1 Fig — Alignments are shown for the A. rpl16 and B. rps16 genes across various Corydalis spp. (TIF) [file pone.0289625.s001.tif]

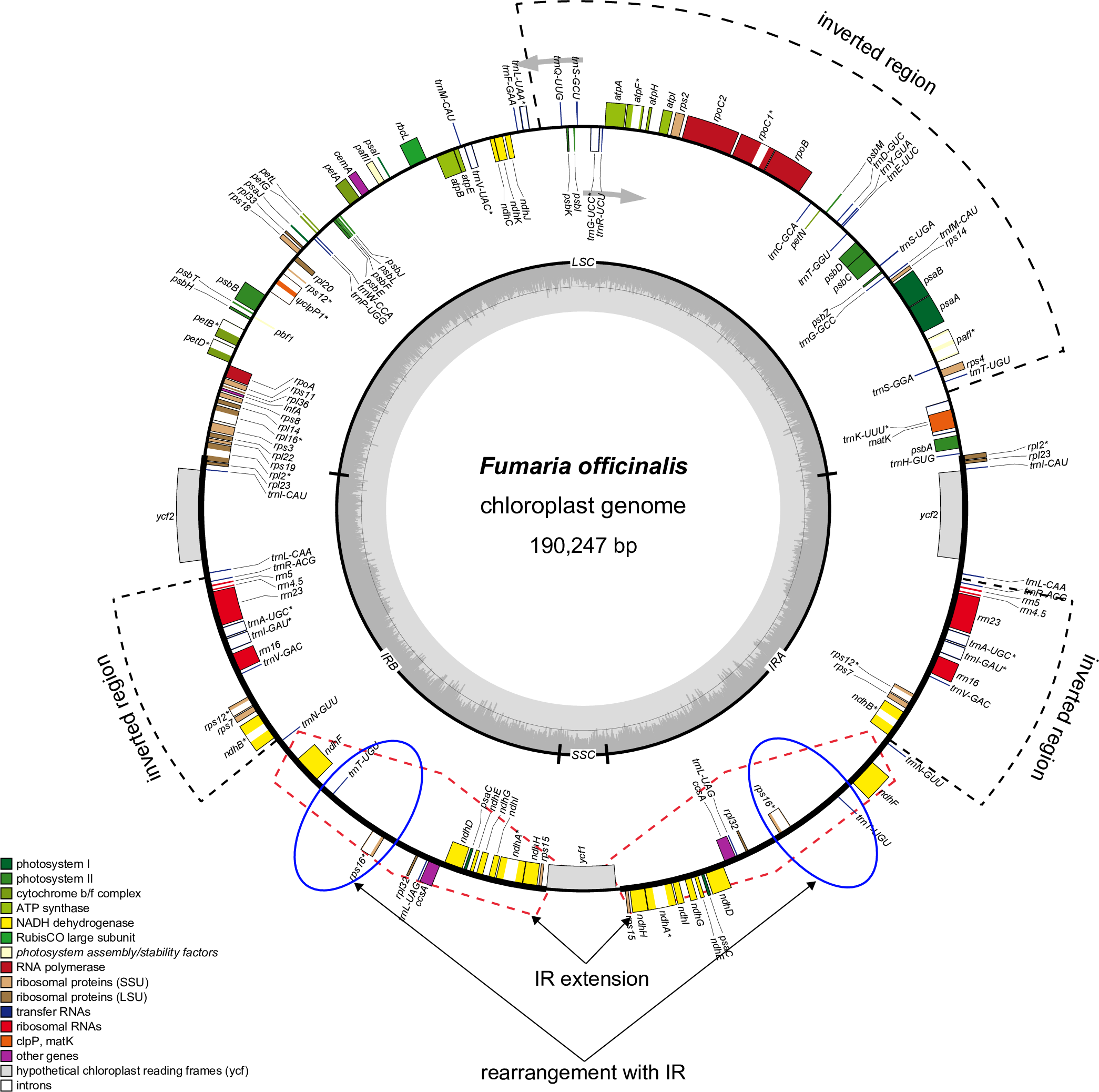

Supplement: S2 Fig — Genes shown outside the circle are transcribed in the counter counter-clockwise direction, and those inside the circle are transcribed in the clockwise direction. The colored bars indicate genes belonging to different functional groups. The inner circles denote the GC content (dark grey) and AT content (light grey) of the genome. The ψ signifies pseudogenes. (TIF) [file pone.0289625.s002.tif]

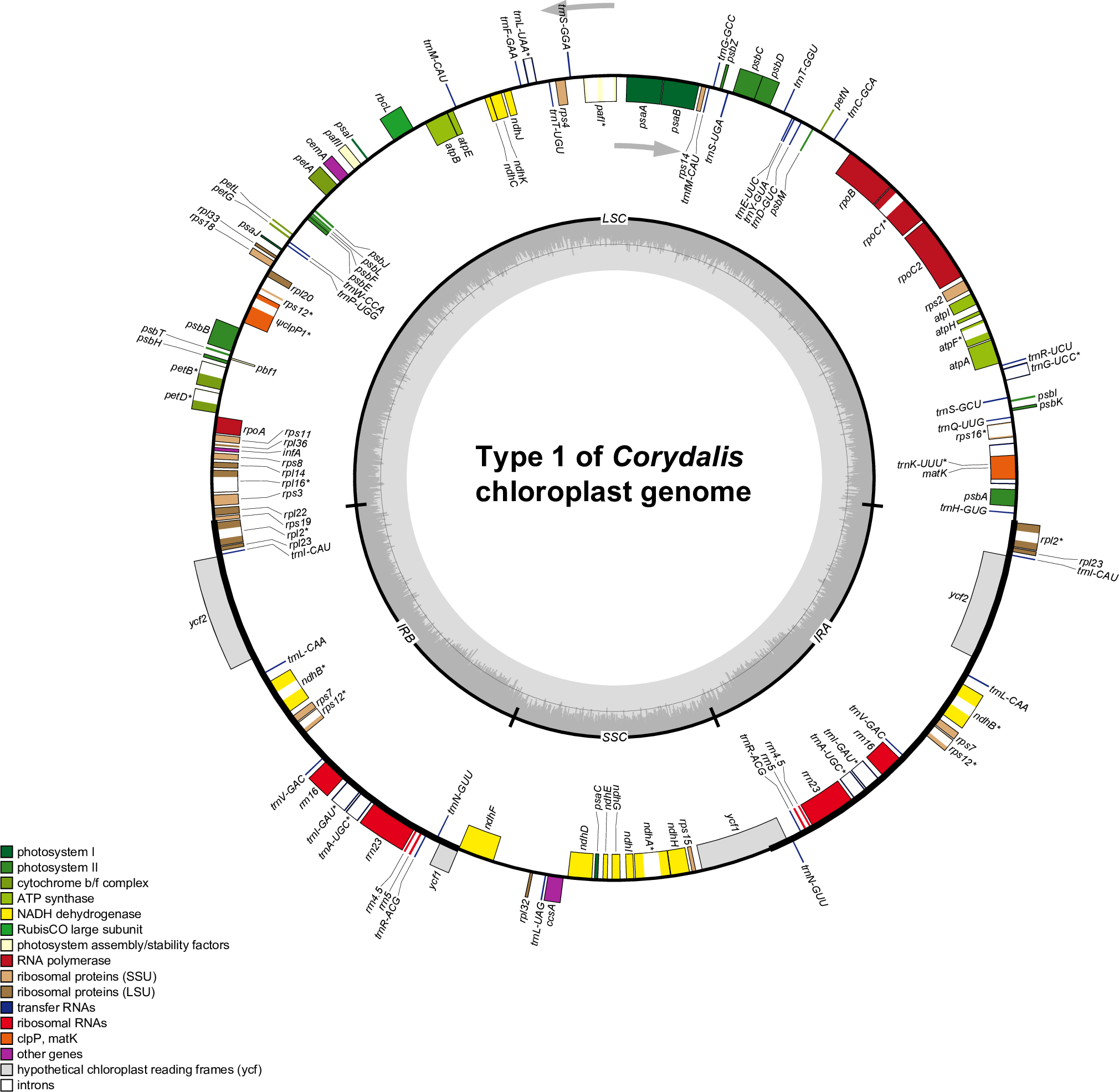

Supplement: S3 Fig — Genes shown outside the circle are transcribed in the counter counter-clockwise direction, and those inside the circle are transcribed in the clockwise direction. The colored bars indicate genes belonging to different functional groups. The inner circles denote the GC content (dark grey) and AT content (light grey) of the genome. The ψ signifies pseudogenes. (TIF) [file pone.0289625.s003.tif]

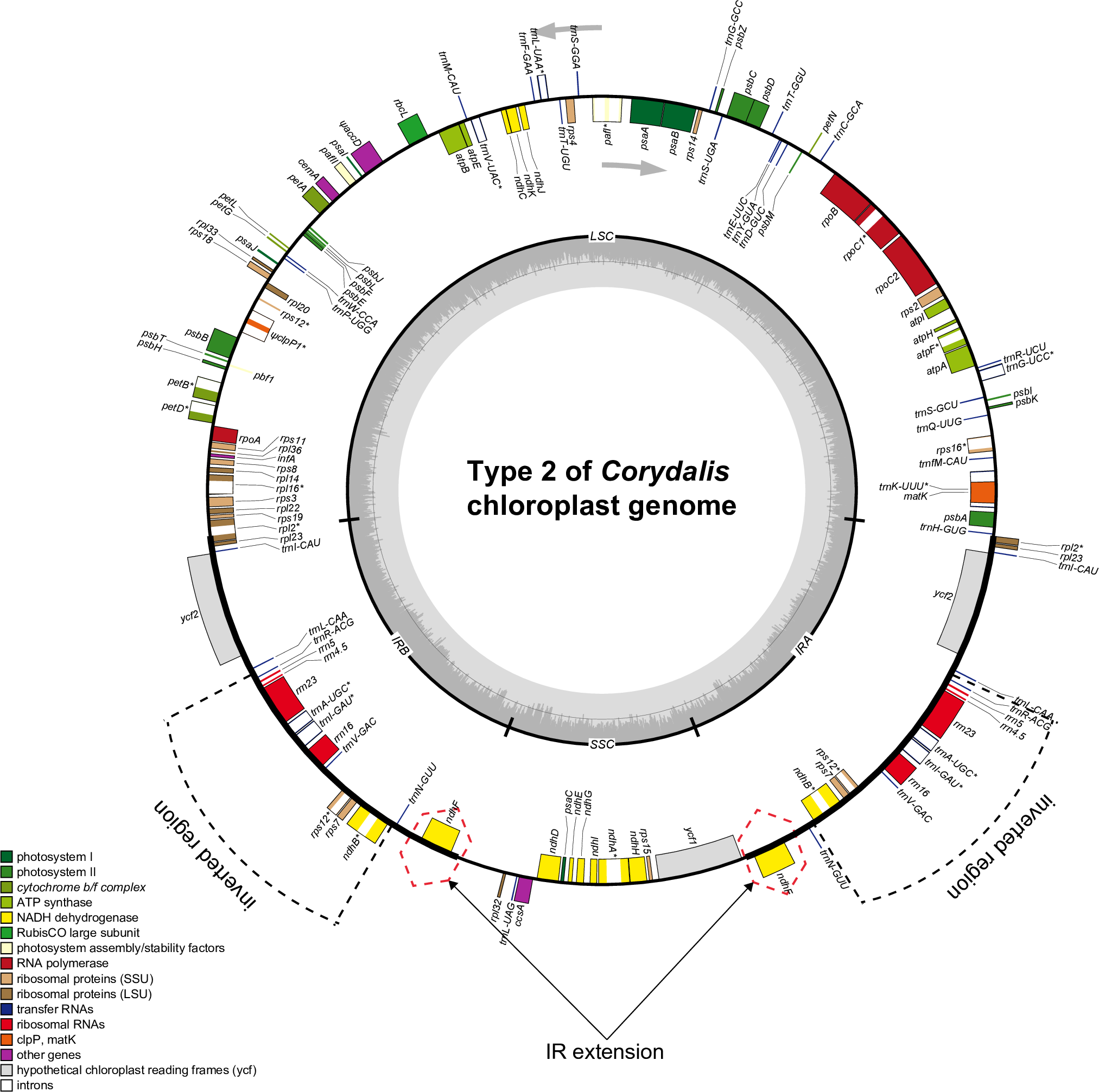

Supplement: S4 Fig — Genes shown outside the circle are transcribed in the counter counter-clockwise direction, and those inside the circle are transcribed in the clockwise direction. The colored bars indicate genes belonging to different functional groups. The inner circles denote the GC content (dark grey) and AT content (light grey) of the genome. The ψ signifies pseudogenes. (TIF) [file pone.0289625.s004.tif]

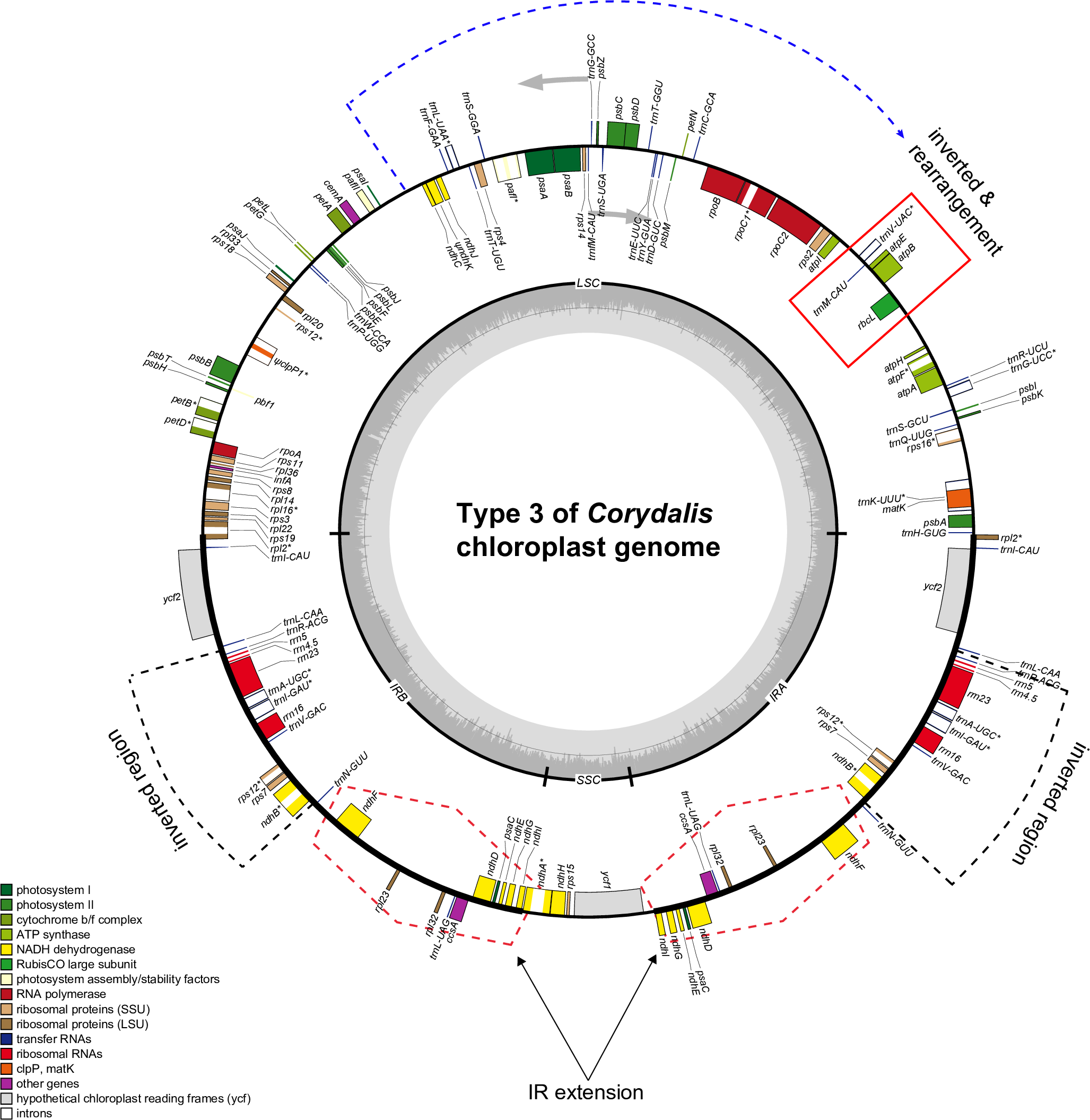

Supplement: S5 Fig — Genes shown outside the circle are transcribed in the counter counter-clockwise direction, and those inside the circle are transcribed in the clockwise direction. The colored bars indicate genes belonging to different functional groups. The inner circles denote the GC content (dark grey) and AT content (light grey) of the genome. The ψ signifies pseudogenes. (TIF) [file pone.0289625.s005.tif]

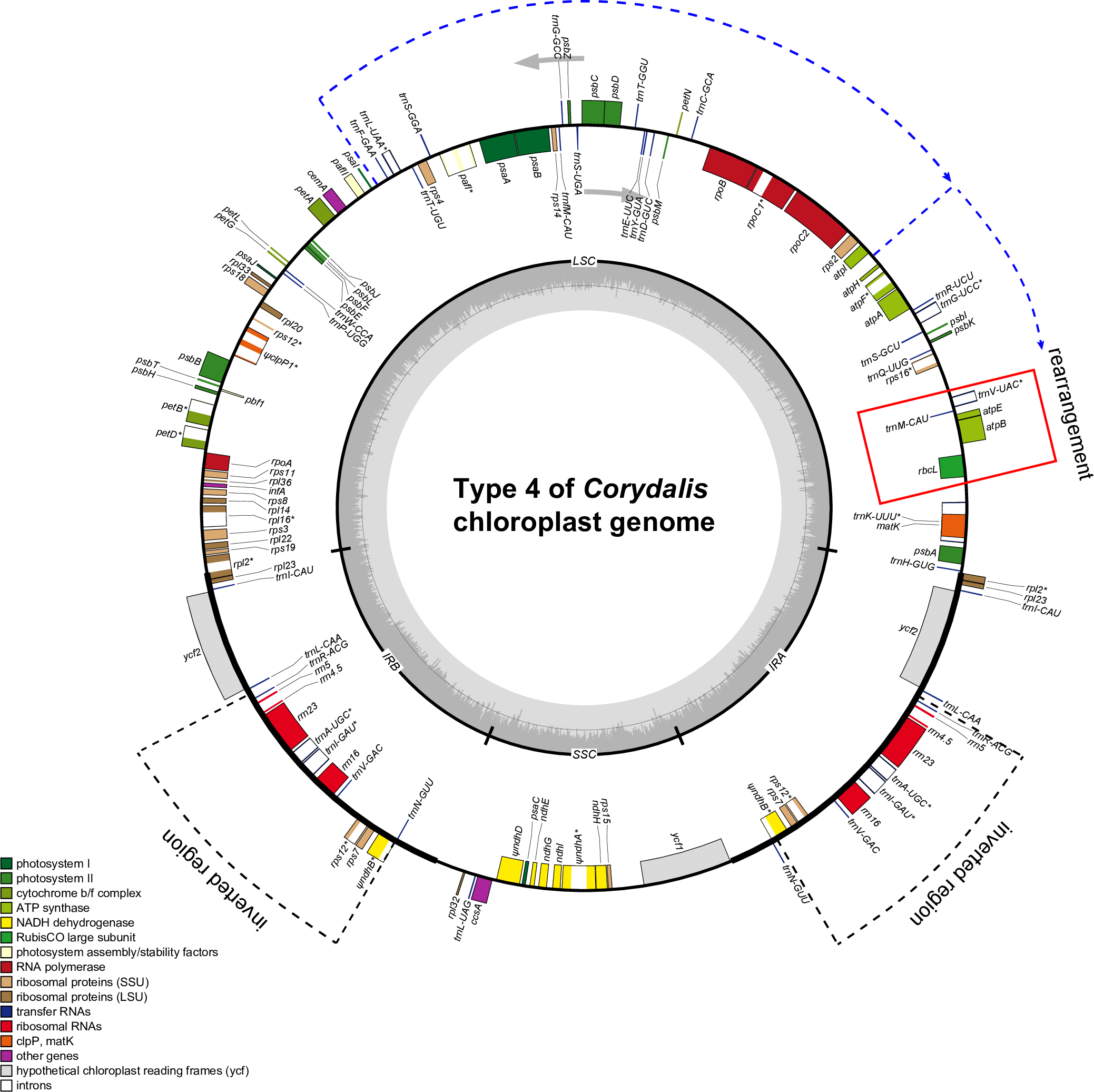

Supplement: S6 Fig — Genes shown outside the circle are transcribed in the counter counter-clockwise direction, and those inside the circle are transcribed in the clockwise direction. The colored bars indicate genes belonging to different functional groups. The inner circles denote the GC content (dark grey) and AT content (light grey) of the genome. The ψ signifies pseudogenes. (TIF) [file pone.0289625.s006.tif]

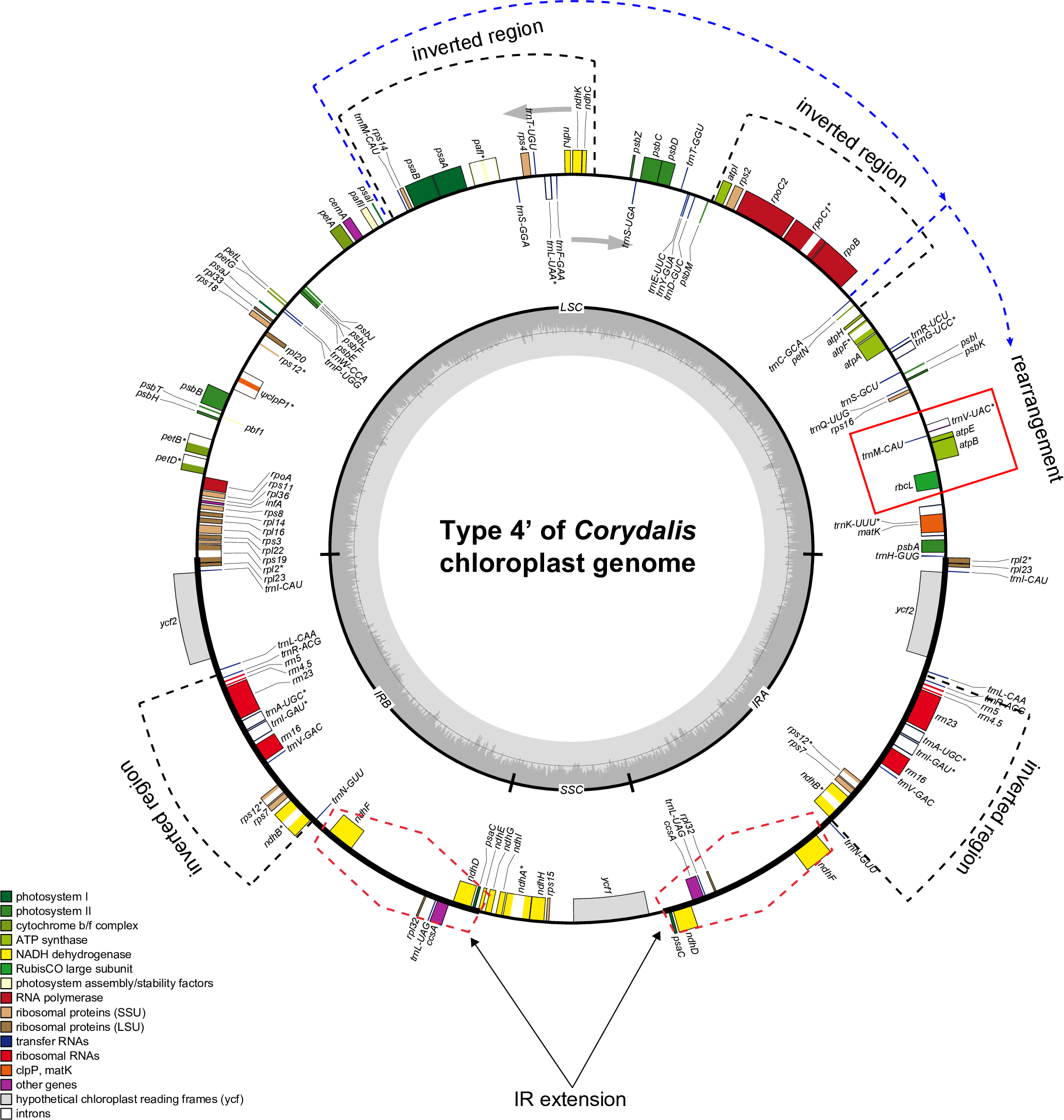

Supplement: S7 Fig — Genes shown outside the circle are transcribed in the counter counter-clockwise direction, and those inside the circle are transcribed in the clockwise direction. The colored bars indicate genes belonging to different functional groups. The inner circles denote the GC content (dark grey) and AT content (light grey) of the genome. The ψ signifies pseudogenes. (TIF) [file pone.0289625.s007.tif]

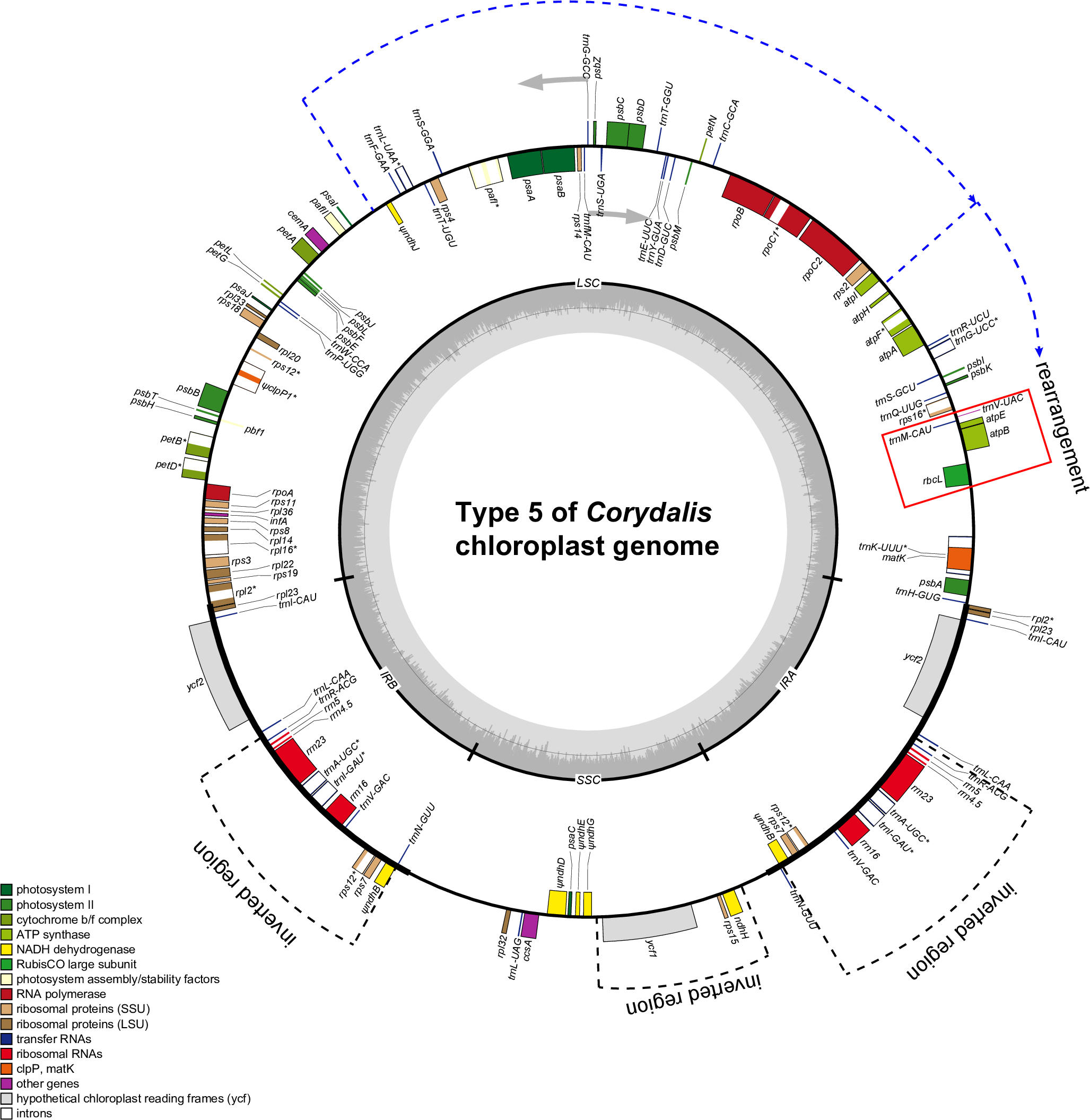

Supplement: S8 Fig — Genes shown outside the circle are transcribed in the counter counter-clockwise direction, and those inside the circle are transcribed in the clockwise direction. The colored bars indicate genes belonging to different functional groups. The inner circles denote the GC content (dark grey) and AT content (light grey) of the genome. The ψ signifies pseudogenes. (TIF) [file pone.0289625.s008.tif]

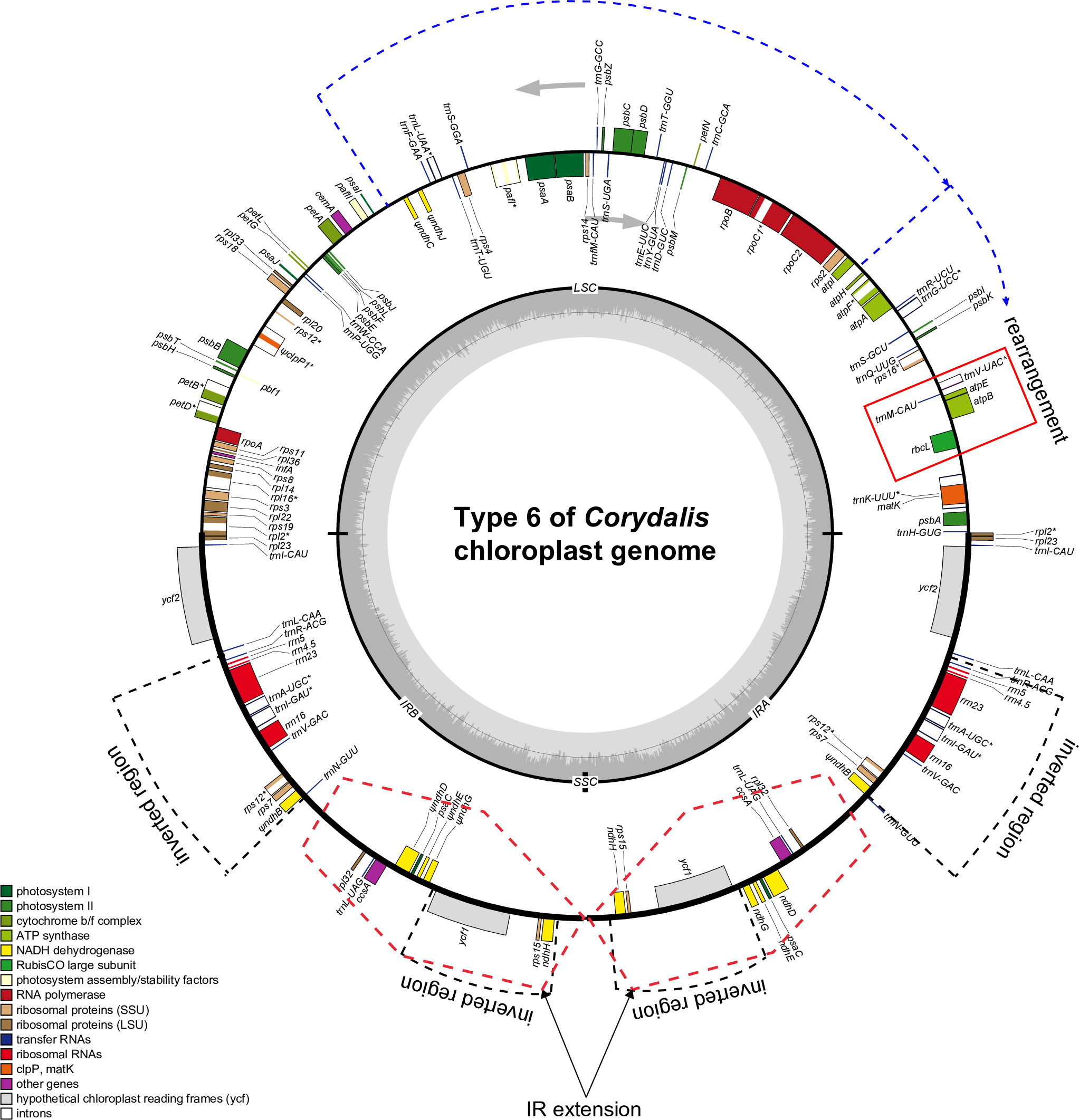

Supplement: S9 Fig — Genes shown outside the circle are transcribed in the counter counter-clockwise direction, and those inside the circle are transcribed in the clockwise direction. The colored bars indicate genes belonging to different functional groups. The inner circles denote the GC content (dark grey) and AT content (light grey) of the genome. The ψ signifies pseudogenes. (TIF) [file pone.0289625.s009.tif]

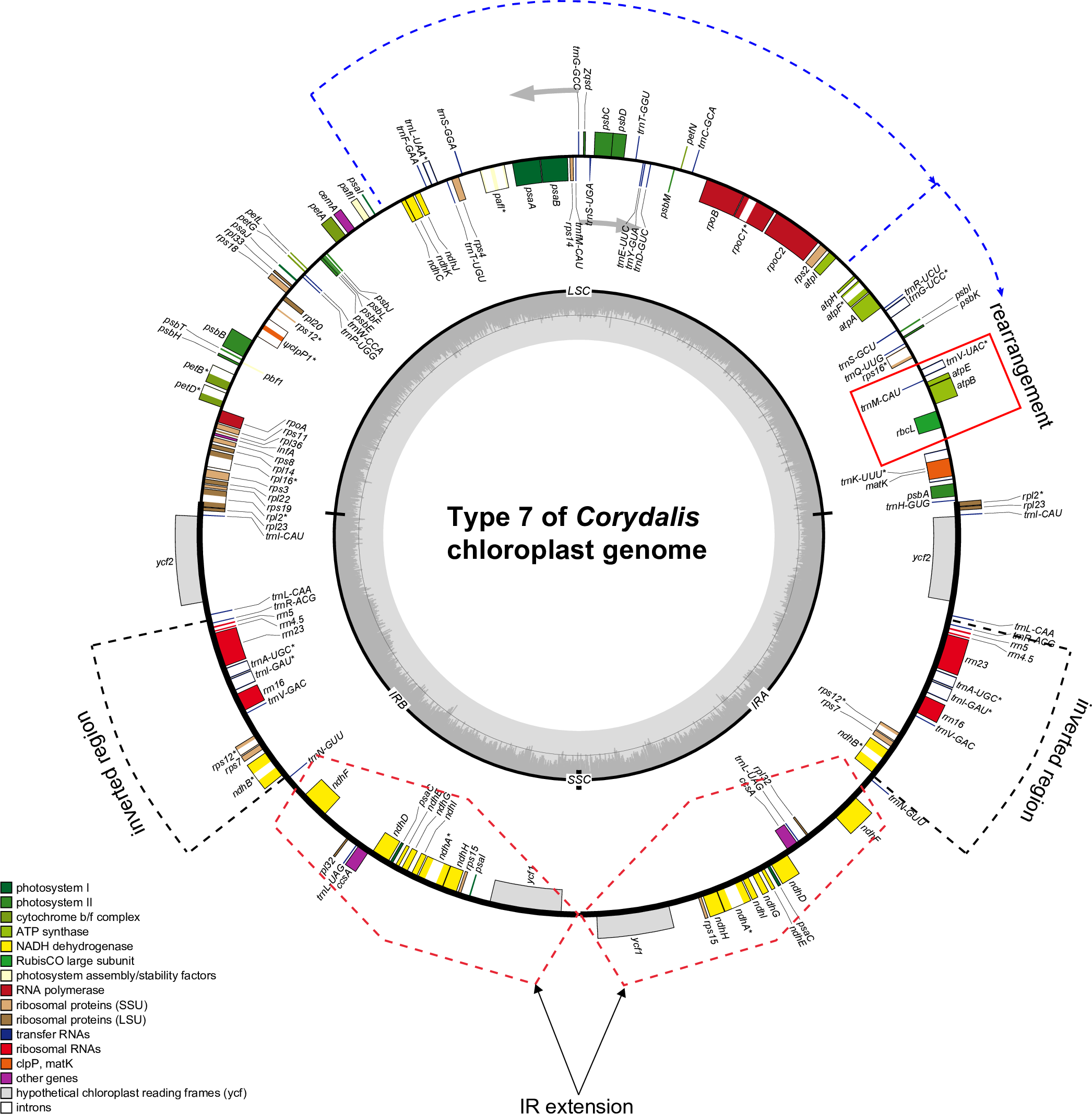

Supplement: S10 Fig — Genes shown outside the circle are transcribed in the counter counter-clockwise direction, and those inside the circle are transcribed in the clockwise direction. The colored bars indicate genes belonging to different functional groups. The inner circles denote the GC content (dark grey) and AT content (light grey) of the genome. The ψ signifies pseudogenes. (TIF) [file pone.0289625.s010.tif]

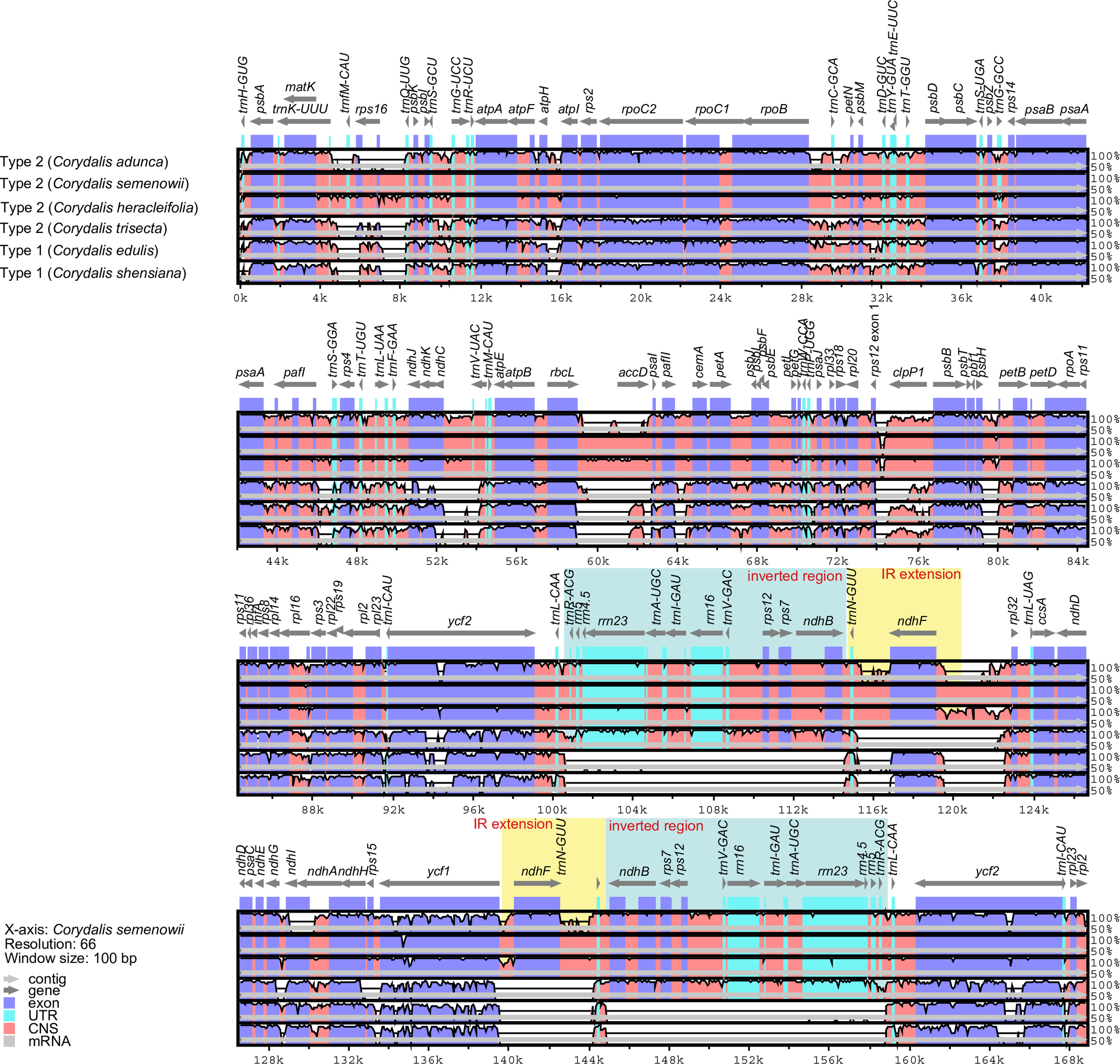

Supplement: S11 Fig — Grey arrows indicate the orientation of genes, red bars represent non-coding sequences, purple bars represent exons, and blue bars represent introns. The vertical scale indicates the percentage identity within 50–100%. (TIF) [file pone.0289625.s011.tif]

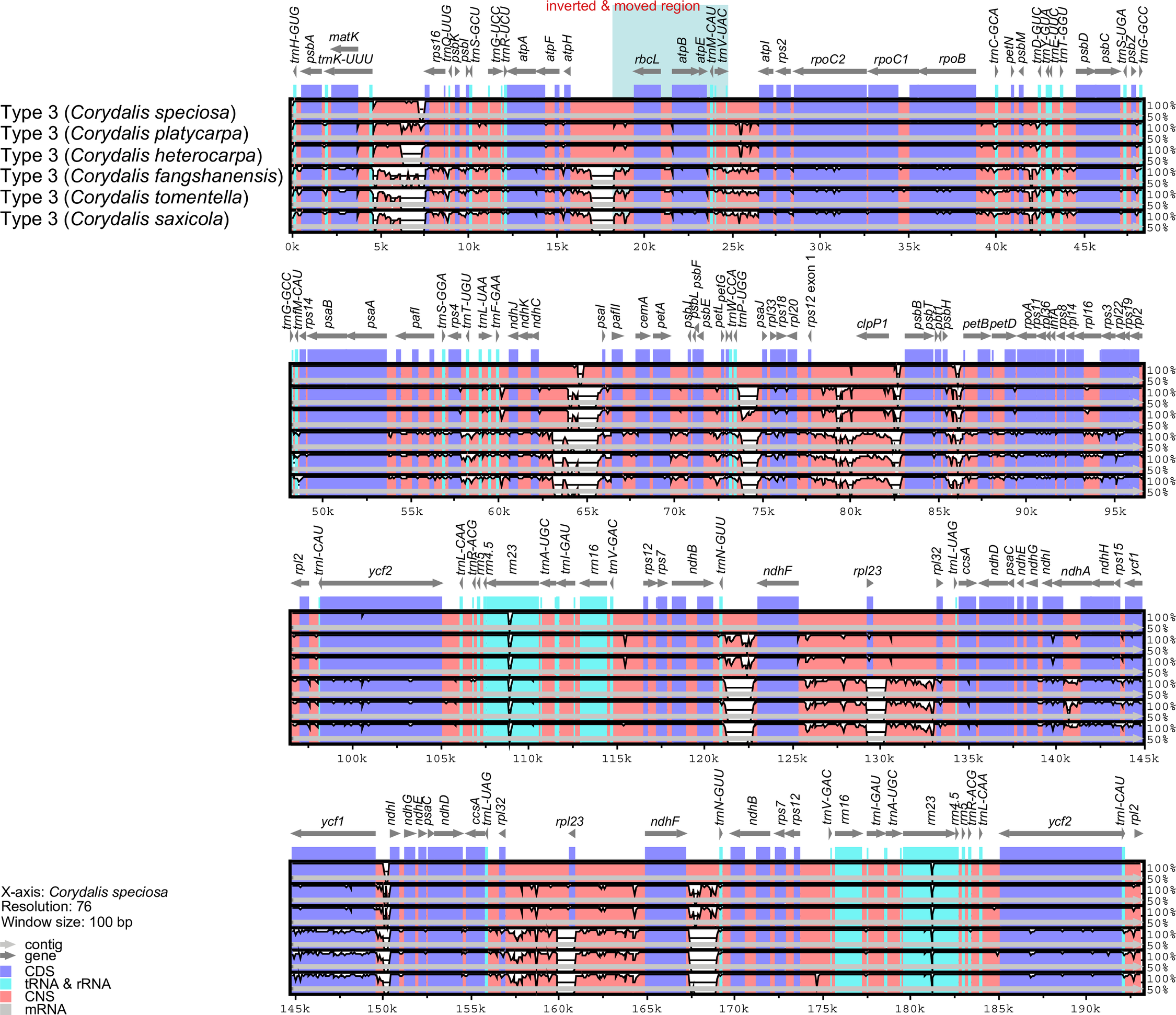

Supplement: S12 Fig — Grey arrows indicate the orientation of genes, red bars represent non-coding sequences, purple bars represent exons, and blue bars represent introns. The vertical scale indicates the percentage identity within 50–100%. (TIF) [file pone.0289625.s012.tif]

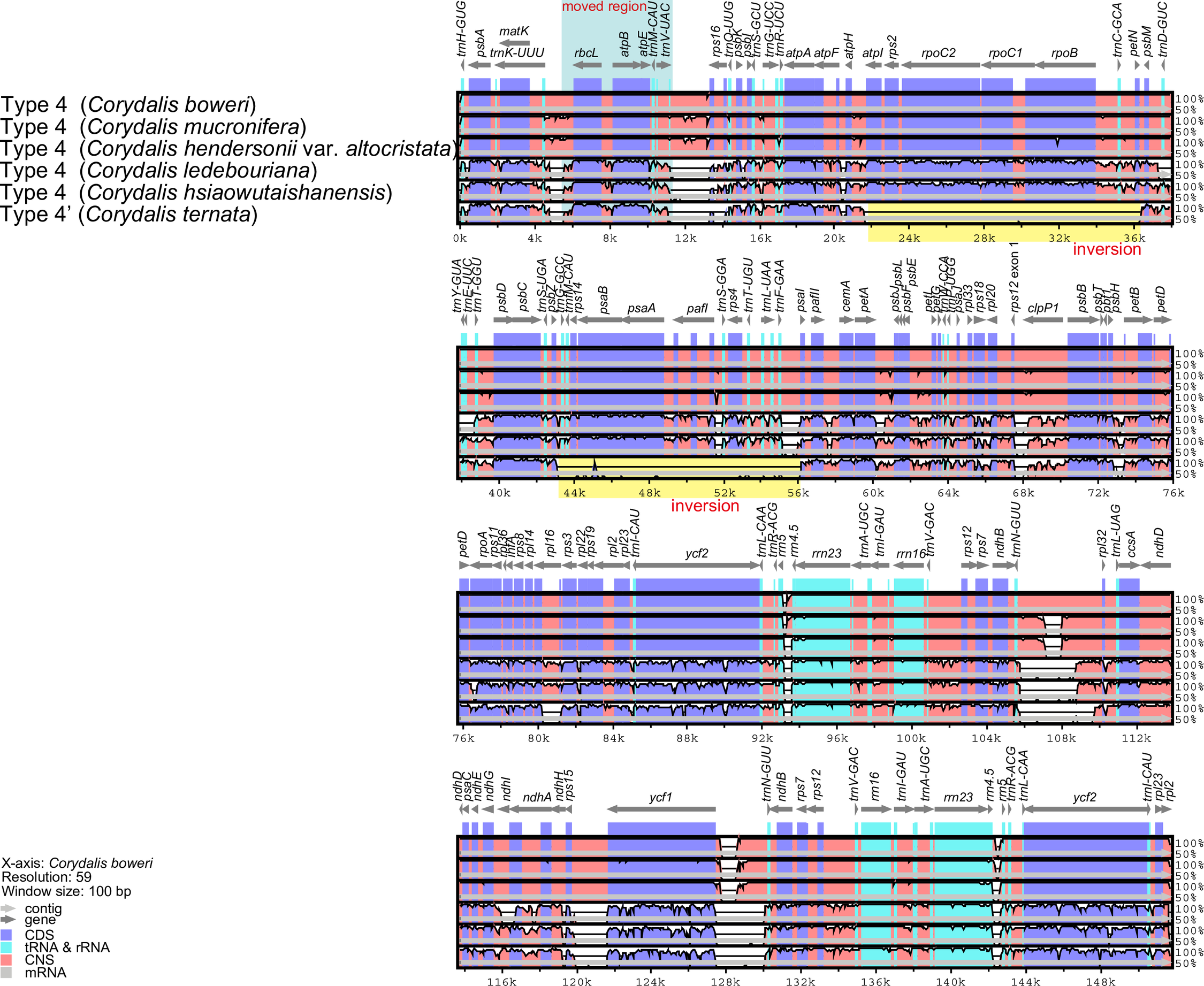

Supplement: S13 Fig — Grey arrows indicate the orientation of genes, red bars represent non-coding sequences, purple bars represent exons, and blue bars represent introns. The vertical scale indicates the percentage identity within 50–100%. (TIF) [file pone.0289625.s013.tif]

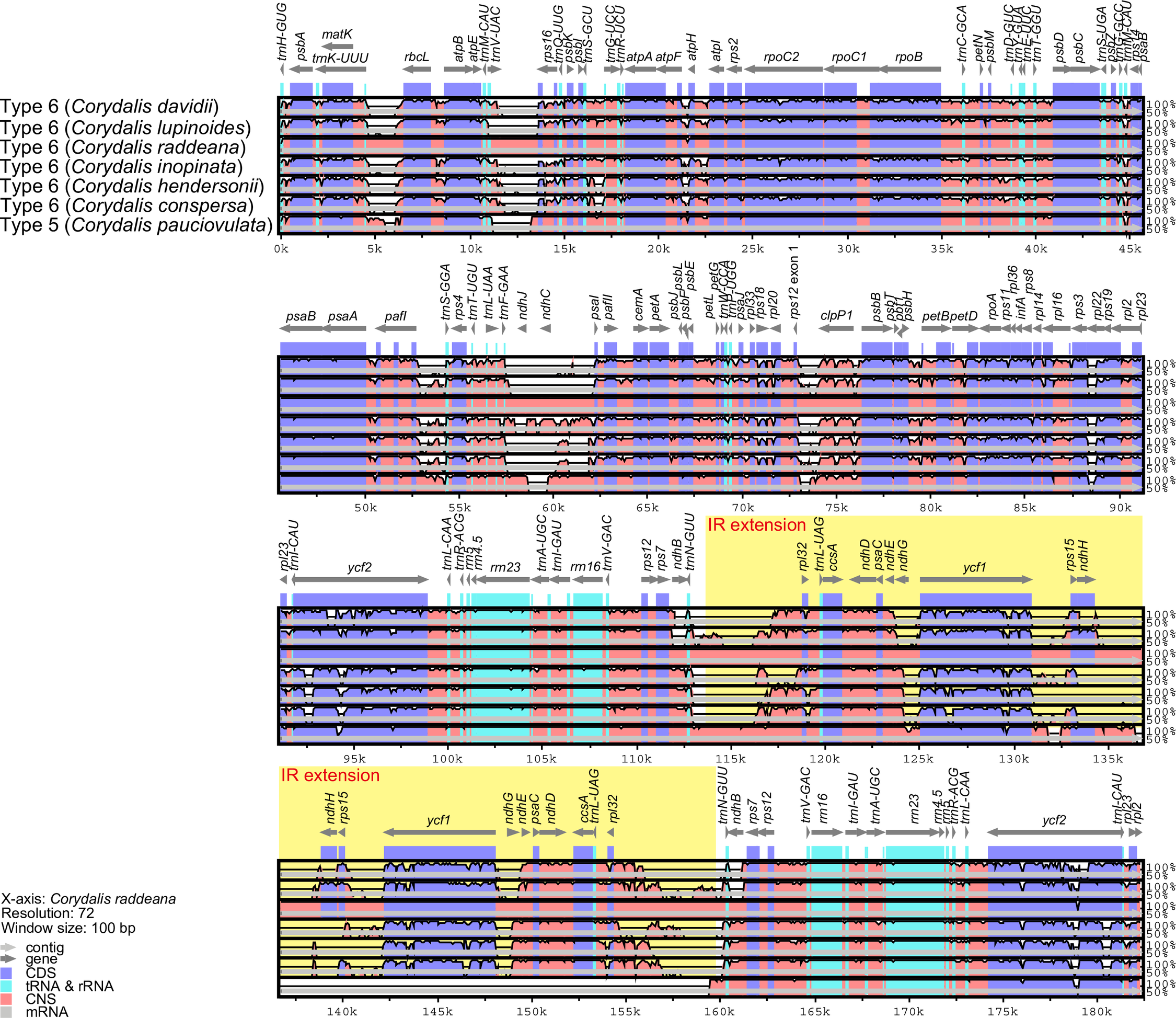

Supplement: S14 Fig — Grey arrows indicate the orientation of genes, red bars represent non-coding sequences, purple bars represent exons, and blue bars represent introns. The vertical scale indicates the percentage identity within 50–100%. (TIF) [file pone.0289625.s014.tif]

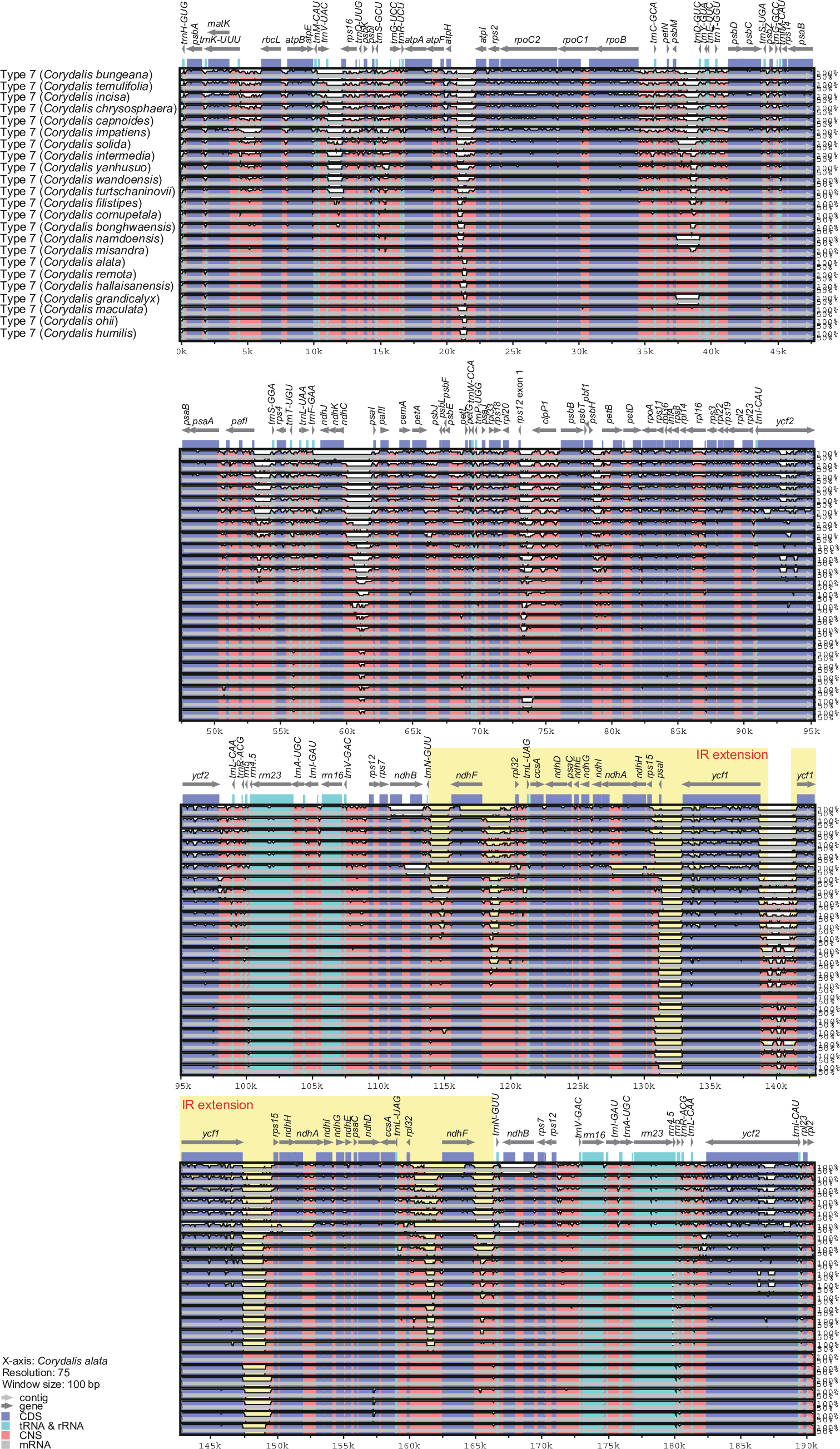

Supplement: S15 Fig — Grey arrows indicate the orientation of genes, red bars represent non-coding sequences, purple bars represent exons, and blue bars represent introns. The vertical scale indicates the percentage identity within 50–100%. (TIF) [file pone.0289625.s015.tif]
