# Supplementary material for: Comparative analysis of the complete chloroplast genome of Papaveraceae to identify rearrangements within the Corydalis chloroplast genome
Source: PLoS One. 2023 Sep 21;18(9):e0289625. doi: 10.1371/journal.pone.0289625 (PMC10513226; doi:10.1371/journal.pone.0289625)
Supplement: Attachment — Submitted filename: PONE-D-23-03735-reviever response.docx [file pone.0289625.s024.docx]

PONE-D-23-03735

In this manuscript (PONE-D-23-03735), authors reported complete chloroplast genomes of 36 *Corydalis* spp. and one *Fumaria* species, and compared these genomes with 22 other taxa’s genomes. The study could be interesting for publication in PLOS ONE. However, the manuscript has some questions which I think need to be addressed.

Major：
1. From Figure 6, we knew some chloroplast genomes were incomplete. The authors should firstly confirm the analyzed 58 chloroplast genomes complete or incomplete. Then, compare the complete chloroplast genomes structure, gene contents, molecular evolution, and so on.

: **All 58 chloroplast genomes we analyzed has complete structures. Figure 6 shows the rearrangement of the genome.**

2. S4 Table and S5 Table should be in the manuscript, not in the supplementary section.

: **We have included two tables in the manuscript based on the reviewer's comments.**

3. There were so many chloroplast genomes of Papaveraceae and *Corydalis*, divergence time estimation may be analyzed and discussed in details.

: **Our thesis was written based on the comparison of the chloroplast genome structure of corydalis, and the amount of papers increased when the branch estimation was analyzed, so the amount of papers was divided for future analysis.**

Minor:

1. There are some repeats of subheadings.

: **Repetition of subheadings is acknowledged. However, a total of nine chloroplast structures were identified, and we tried to explain each one.**
